# Evidence in Lager Yeasts of β-Lyase Activity Breaking Down γ-GluCys-Conjugates More Efficiently Than Cys-Conjugates to Odorant Beer Polyfunctional Thiols

**DOI:** 10.3390/molecules30020325

**Published:** 2025-01-15

**Authors:** Romain Christiaens, Margaux Simon, Raphaël Robiette, Sonia Collin

**Affiliations:** 1 Unité de Brasserie et des Industries Alimentaires, Louvain Institute of Biomolecular Science and Technology (LIBST), Faculté des Bioingénieurs, Université Catholique de Louvain, Croix du Sud, 2 Box L7.05.07, 1348 Louvain-la-Neuve, Belgium; romain.christiaens@uclouvain.be (R.C.); margaux.simon@uclouvain.be (M.S.); 2 Institute of Condensed Matter and Nanosciences (IMCN), Université catholique de Louvain, Place Louis Pasteur, 1348 Louvain-la-Neuve, Belgium; raphael.robiette@uclouvain.be

**Keywords:** beer, aroma, γ-glutamylcysteine-3-sulfanylhexanol, *Saccharomyces pastorianus*, *Saccaromyces cerevisiae* var. *chevalieri*, fermentation, polyfunctional thiol

## Abstract

The prevalence of glutathionylated (G-) precursors of polyfunctional thiols (PFTs) over their free forms has prompted investigating how to optimize the enzymatic breakdown of these precursors with yeast during lager, ale, and non-alcoholic/low-alcoholic beer (NABLAB) fermentation trials. Some *Saccharomyces cerevisiae* yeasts have been selected for their higher β-lyase activity on the cysteinylated (Cys-) conjugates (up to 0.54% for SafAle^TM^ K-97), yet some *S. pastorianus* strains and one maltose-negative *S. cerevisiae* var. *chevalieri* yeast have proved to release PFTs more efficiently from G-precursors (up to 0.21% for BRAS-45 and 0.19% for SafBrew^TM^ LA-01). The present study aimed to explore the possibility and extent of direct release in the beer of 3-sulfanylhexanol from its synthetic γ-glutamylcysteinylated (γ-GluCys-) precursor. Release efficiency was determined by GC-PFPD after the fermentation (7 days at 24 °C and 3 days at 4 °C) of a 15 °Plato (°P) wort enriched with 15 mg/L of synthesized γ-GluCys-3SHol. Up to a 0.28–0.35% release was measured with *S. pastorianus* strains BRAS-45 and SafLager^TM^ E-30, while much lower activities (≤0.16%) were observed with *S. cerevisiae* yeasts, including the maltose-negative *chevalieri* variety. This β-lyase activity on γ-GluCys-3SHol has never been described before. Under our experimental conditions, the efficiency of release from γ-GluCys-3SHol was drastically reduced in low-density worts. A strongly strain-dependent impact of temperature was also observed.

## 1. Introduction

Among other essential oils, polyfunctional thiols (PFTs) have emerged over the last decade as key flavor contributors of most fermented beverages [1,2,3,4,5,6], especially white wines and dry-hopped beers. Among them, 3-sulfanylhexanol (3SHol, with a nice passion fruit aroma and perception threshold of 2.5 ng/L for the S-enantiomer [7]) was found in almost all grape [7,8] and hop [6] cultivars. In beer, polyfunctional thiols enhance the aromatic profile by adding complexity and contributing, along with esters and terpenes, to citrus and tropical fruit aromas characteristic of dual-purpose hop varieties [6].

During fermentation, the breakdown by wine or brewing yeasts of S-conjugate precursors significantly increases the quantity of free PFTs. Both glutathionylated (G-) and cysteinylated (Cys-) precursors are formed in grapes and hops through detoxification pathways (e.g., G-3SHol arises through the addition of glutathione onto *trans*-2-hexenal, a toxic aldehyde resulting from linolenic acid oxidation) [9,10,11]. For instance, up to 100 mg/kg G-3SHol was found in the Amarillo hop variety, while only less than 1 µg/kg was detected as free form [10]. Even malt appears as a significant source of glutathionylated precursors [12].

Various studies highlighted the role of yeast, particularly *Saccharomyces* and non-*Saccharomyces* strains, in releasing aromatic thiols during wine fermentation [13]. Others have explored the enzymatic mechanisms responsible for the release of PFTs in beer through primary [14,15], secondary [16], and even bottle fermentation [17].

When starting from Cys-precursors, *S. cerevisiae* uses its β-lyase activity to release sulfanylalkyl alcohols and aldehydes [18]. The subsequent esterification of the sulfanylalkyl alcohols produced can lead to, for instance, 3-sulfanylhexyl acetate (3SHA), which is even more odorant than its alcohol counterpart [19]. Among various tested brewing ale yeasts, SafAle^TM^ K-97 was recently shown to exhibit the highest β-lyase activity, reaching up to 0.54% release from Cys-3SHol. Yeast SafAle^TM^ S-33 showed superior acetyl-transferase activity, esterifying up to 80% of the alcohols released, especially from Cys-3-sulfanylpentanol (Cys-3SPol) and Cys-3-sulfanyl-4-methylpentanol (Cys-3S4MPol) [14].

**Figure 1:**
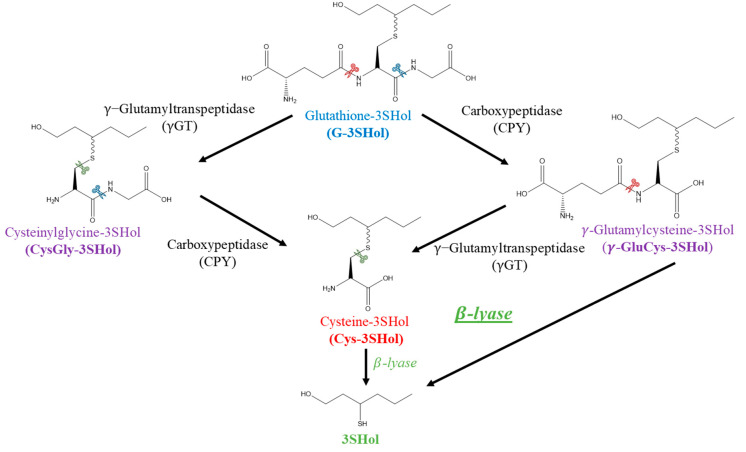
Potential biodegradations of 3-sulfanylhexanol (3SHol) G-precursors.

For these brewing ale yeasts, the efficiency of release from G-precursors proved to be much lower (under 0.08%). To degrade G-precursors, a multi-step pathway leading to Cys-counterparts was proposed, requiring both carboxypeptidase (CPY) and γ-glutamyltranspeptidase (γ-GT) (Figure 1). In wine fermentation trials involving *S. cerevisiae*, it has been demonstrated that the γ-GluCys intermediate is formed from G-precursors [20]. This shows that yeast can cleave the amide bond between cysteine and glycine [21]. On the other hand, no production of the CysGly- intermediate from the G-adduct was observed, which suggests that γ-GT activity is not involved in the PFTs release [22]. In *S. cerevisiae*, γ-GT has been identified as a vacuolar enzyme, playing a role in amino acid transport [18,23,24]. One cannot exclude that some yeast strains might use it to degrade the tripeptide, as is carried out for malt enzymes during beer wort preparation. Chenot et al. evidenced, during mashing, the formation of the CysGly-intermediate, which attests to potent γ-GT activity in barley malt (optimal pH: 5.4) [25]. If not degraded during brewing, this intermediate could also be consumed by yeast during fermentation.

Recently, some *S. pastorianus* strains have been shown to display higher release of PFTs from G-adducts (achieving rates of 0.21% from G-3SHol with BRAS-45) than from Cys-adducts. These results exclude the multi-step pathway involving β-lyase activity on Cys-conjugates only [15]. Likewise, the maltose-negative strain SafBrew^TM^ LA-01 has recently emerged as an even more potent degrader of G-3SHol to free thiols, despite being classified as an *S. cerevisiae* strain [26]. This is of particular interest in the aim of using it for producing pleasant non-alcoholic or low-alcoholic beers (NABLABs) with more fruity fermentation aromas (the main defect of commercial NABLABs, together with persistent methional and sotolone taints [27,28]).

The aim of the present study was to explore whether *S. pastorianus* strains and *S. cerevisiae* var. *chevalieri* SafBrew^TM^ LA-01 might still better use γ-GluCys-conjugates as substrates. These conjugates have recently been shown to predominate when special malts are used because of cysteine–glycine bond breakdown at high kilning temperature [12]. Moreover, yeast carboxypeptidase could create them from glutathione during fermentation. Here, 3SHol amounts released from γ-GluCys-3SHol were determined for eleven strains (including SafAle^TM^ S-33, SafAle^TM^ K-97, and SafBrew^TM^ LA-01) in a 15 °P wort spiked with the conjugate. To allow us to compare our results with previously published data, all experiments were first conducted for 7 days at 24 °C, followed by 3 days at 4 °C. In a second experiment, the impact of temperature was assessed for the two best γ-GluCys-3SHol metabolizing yeast candidates. Lastly, with a view to produce pleasant 3SHol and 3SHA in NABLABs, the impact of using a 6 °P wort was determined for three yeasts, including the maltose-negative *chevalieri* variety.

## 2. Results and Discussion

### 2.1. Release of Free 3SHol from γ-GluCys-3SHol in Beer Fermentation Trials

To assess the efficiency of 3SHol release from γ-GluCys-3SHol by eight *S. pastorianus* strains and one maltose-negative strain (*S. cerevisiae* var. *chevalieri* SafBrew^TM^ LA-01), we first used the same fermentation conditions as previously applied by Chenot et al. to investigate G- and Cys-3SHol degradation (15 °P initial wort density, 7 days of fermentation at 24 °C, 3 days of maturation at 4 °C) [15], even though the brewing industry habitually uses lager yeasts at lower temperatures. All results were compared to the performances of SafAle^TM^ K-97 *S. cerevisiae* (exhibiting the best release efficiency among the previously tested ale yeasts) and SafAle^TM^ S-33 (exhibiting the best acetylation efficiency among the previously tested yeasts) [14,15].

**Figure 2:**
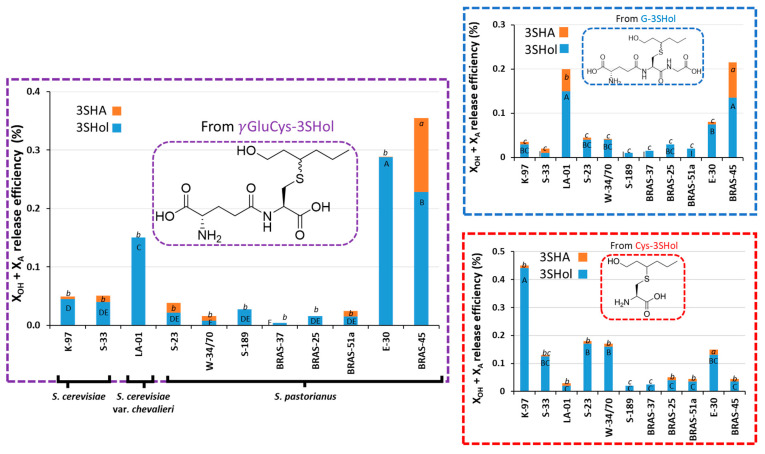
Capacity of lager and ale yeasts to yield 3SHol and 3SHA from γ-GluCys-3SHol at 24 °C, alongside comparisons with previous data on the release from G- and Cys-3SHol. Values with different letters are significantly different (*p* < 0.05) according to the Student–Newman–Keuls test (between duplicate variation coefficients below 15%) [14,15,26].

As depicted in Figure 2, the lager yeasts BRAS-45 and SafLager^TM^ E-30 reached the highest efficiencies of release from the dipeptidic precursor: 0.35% and 0.28%, respectively. These values were much higher than those obtained for Cys-3SHol (0.03% and 0.02%). This suggests the existence of β-lyase activity directly targeting the dipeptidic precursor, without any requirement for γ-GT activity. On the other hand, both values were above those obtained for G-3SHol (0.21% and 0.08%, respectively). This seems logical, as the pathway from G-3SHol to free thiol probably involves one more step. Our data suggest that some lager yeast strains display this new kind of β-lyase activity breaking down γ-GluCys-conjugates more efficiently than Cys-conjugates to odorant polyfunctional thiols.

The rates of esterification by BRAS-45 were similar (about 30%), whether γ-GluCys- or G-precursors were used. This makes BRAS-45 the most promising strain in terms of aromatic potential (lower threshold and better descriptor for 3SHA than for 3SHol). The yeast SafAle^TM^ K-97 exhibited the lowest efficiency of release from γ-GluCys-3SHol, in keeping with the release rates observed with G-precursors.

Regarding SafBrew^TM^ LA-01, this *S. cerevisiae* var. *chevalieri* yeast was still more efficient at processing the G-adduct (0.19%) than the γ-GluCys-intermediate (0.15%). This suggests that the action of its β-lyase can overcome the steric hindrance of glutathione and directly release thiol from it.

### 2.2. Impact of Fermentation Temperature

**Figure 3:**
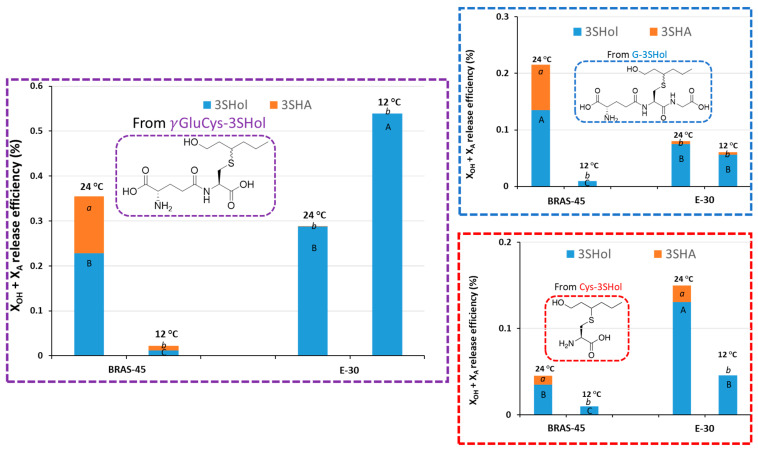
Impact of temperature on the release, by the lager yeasts BRAS-45 and E-30 (15 °P wort), of 3SHol from G-, γ-GluCys-, and Cys-3SHol. Values with different letters are significantly different (*p* < 0.05) according to the Student–Newman–Keuls test (between duplicate variation coefficients below 15%).

In considering their outstanding efficiency of release from γ-GluCys-conjugates at 24 °C, *S. pastorianus* BRAS-45 and SafLager^TM^ E-30 were further studied for release from G-, γ-GluCys-, and Cys-3SHol at 12 °C, with a more usual fermentation temperature for a lager yeast, and with other parameters kept constant (Figure 3). Lowering the fermentation temperature significantly decreased (up to ten-fold) the release from γ-GluCys-3SHol by BRAS-45. Such a decrease in β-lyase activity has been mentioned previously and is confirmed here on both G-, γ-GluCys-and Cys-3SHol [15].

With SafLager^TM^ E-30, in contrast, release from γ-GluCys-3SHol (but not from G- or Cys- 3SHol) was much greater at 12 °C than at 24 °C. This unexpected result indicates that the impact of temperature is strongly strain-dependent. Deeper investigation at intermediate temperatures might help to further increase hop flavors in lager beers.

### 2.3. Impact of Wort Density

When producing NABLABs with maltose-negative strains, it is usually recommended to start with a 6 °P wort, ensuring an alcohol content under 0.5% (*v*/*v*). In our next experiment, the maltose-negative strain SafBrew^TM^ LA-01 was therefore tested in a 6 °P wort spiked with γ-GluCys-3SHol and compared with our two thiol super-producers BRAS-45 and SafLager^TM^ E-30.

Wort density is known to affect yeast vitality and fermentation metabolites. Chenot et al. observed the best rate of release from Cys-3SHol in a 12 °P wort, while esterification to 3SHA was higher at 18 °P.

**Figure 4:**
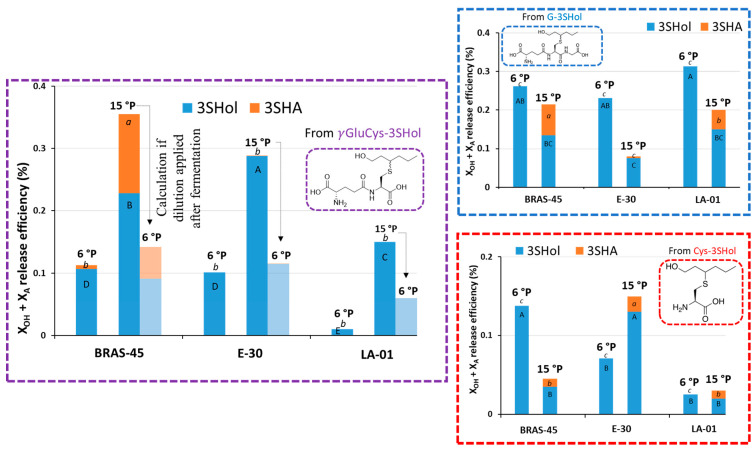
Impact of decreasing the wort density to 6 °P on the release of 3SHol by the lager yeasts BRAS-45 and SafLager^TM^ E-30, and by *S. cerevisiae* var. *chevalieri* SafBrew^TM^ LA-01 (at 24 °C), from G-, γ-GluCys-, and Cys-3SHol. Values with different letters are significantly different (*p* < 0.05) according to the Student–Newman–Keuls (between duplicate variation coefficients below 15%).

As depicted in Figure 4, wort dilution to 6 °P prior to fermentation led to a drastic loss of esterification activity, whatever the strain or the precursor, confirming previously observed trend.

Moreover, in the 6 °P wort, only 0.1% was released by BRAS-45 and SafLager^TM^ E-30 from γ-GluCys-3SHol (versus 0.28–0.35% in the 15 °P trials). With SafBrew^TM^ LA-01, release was also reduced to only 0.01% (this represents a 15-fold decrease compared to 15 °P). Our data indicate that dilution after fermentation (calculated release of 0.06%) could be preferable with this maltose-negative strain for better use of γ-GluCys-precursors in NABLABs.

On the other hand, working at 6 °P increases, for all three strains, 3SHol release from the tripeptidic precursor (the most abundant in hop): 0.23–0.31% against 0.08–0.21%. Regarding the utilization of Cys-3SHol, the impact of density remains more strain-dependent.

## 3. Materials and Methods

### 3.1. Chemicals

*N*-Boc-l-glutamic-acid α-*tert*-butyl ester, *N*,*N*-dimethylformamide, benzotriazole-1-yl-oxy-tris-(dimethylamino)-phosphonium hexafluorophosphate (BOP), *N*,*N*-diisopropylethylamine (DIEA), 1,4-dithioerythritol, triethylamine (Et_3_N), dichloromethane (DCM), *trans*-2-h exenal, cesium carbonate, 1,4-dioxane, sodium borohydride, triisopropylsilane (TIS), 6 mL Discovery Ag-ion SPE tubes, 6-sulfanyl-hexanol (6SHol), 3-sulfanylhexanol (3SHol), and 3-sulfanylhexyl acetate (3SHA) were purchased from Sigma-Aldrich (Hoeilaart, Belgium). Absolute ethanol, cyclohexane, ethyl acetate, formic acid, and citric acid monohydrate were purchased from VWR (Leuven, Belgium). Sodium hydrogen carbonate, sodium chloride, sodium sulfate, and magnesium sulfate were purchased from Carl Roth (Karlsruhe, Germany). l-cystine bis (*tert*-butyl ester) dihydrochloride was obtained from A2B Chem (San Diego, CA, USA). Milli-Q water was used (Millipore, Bedford, MA, USA).

### 3.2. Yeasts

Four lager yeasts (BRAS-25, BRAS-37, BRAS-45, and BRAS-51a; propagation in liquid yeast extract/peptone/sucrose (YPS) media at 28 °C) from the INBr UCLouvain collection (Louvain-la-Neuve, Belgium) and seven active dry yeasts (SafAle ^TM^ S-33, SafAle^TM^ K-97, SafLager^TM^ S-23, SafLager^TM^ W34-70, SafLager^TM^ S-189, SafLager^TM^ E-30, and SafBrew^TM^ LA-01) from Fermentis Lesaffre (Marcq-en-Baroeul, France) were used for fermentation trials. The YPS aqueous medium was composed of MgCl_2_ (0.58 g/L), NaCl (0.5 g/L), (NH_4_)_2_SO_4_ (1 g/L), CaCl_2_·2H_2_O (0.1 g/L), KH_2_PO_4_ (1875 g/L), maltose syrup (0.017 g/L), sucrose (108.33 g/L), yeasts extract (18.33 g/L), tryptone (2 g/L), tween 80 (0.1 g/L), and FeCl (1 g/L).

### 3.3. Synthesis of γ-GluCys-3SHol

**Figure 5:**
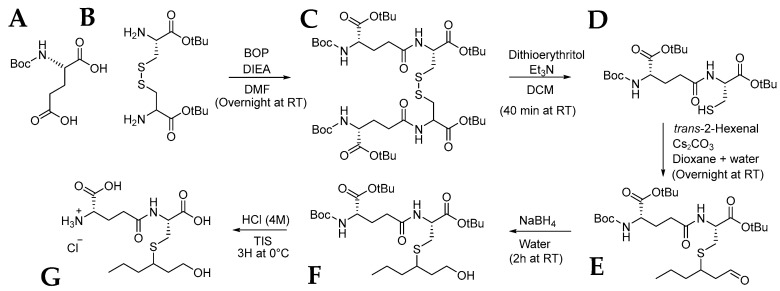
Multi-step organic synthesis of γ-GluCys-3SHol (**G**). *N*-Boc-l-Glutamic acid α-tert-butyl ester (**A**), l*-*cystine di-*tert*-butyl ester dihydrochloride (**B**), Bop, DIEA, DMF, overnight at 25 °C; dithioerythritol, ET_3_N, DCM, 40 min at 25 °C; *trans*-hexenal, CS_2_CO_3_, dioxane, water, overnight at 25 °C; NaBH_4_, water, 2 H at 25 °C; HCl, TIS, 3H at 0 °C.

The multi-step synthesis of γ-GluCys-3SHol was inspired by previous work performed by Bonnaffoux et al. (Figure 5) [20].

#### 3.3.1. Synthesis of *N*-(*N*-Boc-l-γ-glutamyl)-l-cystine Di-*tert*-butyl Ester (**C**)

To a solution of *N*-Boc-l-glutamic-acid α-*tert*-butyl ester (**A**; 1 eq., 20 mmol, 6.067g) dissolved in DMF (200 mL), BOP (1.1 eq., 22 mmol, 9.730 g) was added followed by the addition of DIEA (3.92 eq., 78.4 mmol, 13.66 mL) and L-cystine bis (*tert*-butyl ester) dihydrochloride (**B**; 0.55 eq., 11 mmol, 4.680 g). The reaction mixture was stirred overnight at room temperature, concentrated under reduced pressure before adding ethyl acetate (300 mL). The organic solution was washed three times with 300 mL of KHSO_4_ 1M, one time with a saturated NaCl solution, three times with saturated NaHCO_3_, and one time with saturated NaCl. The solution was dried over MgSO_4_ and concentrated under reduced pressure to obtain a crude mixture (9.28 g), which was purified by flash chromatography on silica gel (cyclohexane/ethyl acetate = 5/5, *v*/*v*) to give a slightly yellow foam product (6.43 g). The structure was confirmed by UPLC-ESI-MS and by ^1^H NMR (300 MHz, CDCl_3_).

#### 3.3.2. Synthesis of *N*-(*N*-Boc-l-γ-glutamyl)-l-cysteine *tert*-Butyl Ester (**D**)

To a solution of *N*-(*N*-Boc-l-γ-glutamyl)-l-cystine bis(*tert*-butyl ester) (**C**; 1 eq., 6.965 mmol, 6.430 g) in DCM (300 mL), Et_3_N (3 eq., 20.895 mmol, 2.912 mL) and dithioerythritol (1.5 eq, 10.448 mmol, 1.61 g) were added under an argon atmosphere. The reaction mixture was stirred for 40 min and was quenched with an aqueous citric acid 5% solution (300 mL). The organic phase was washed twice with 300 mL citric acid 5%, and one time with 300 mL of a NaCl saturated solution. The solution was then dried over MgSO_4_ and concentrated under reduced pressure to afford 7.23 g of crude product. The structure was confirmed by UPLC-ESI-MS and by ^1^H NMR (300 MHz, CDCl_3_).

#### 3.3.3. Synthesis of *S*-3-(Hexan-1-ol)-*N*-(*N*-Boc-l-γ-glutamyl)-l-cysteine *tert*-Butyl Ester (**F**)

To a solution of *N*-(*N*-Boc-l-γ-glutamyl)-l-cysteine *tert*-butyl ester (**D**; 1 eq., 9.92 mmol, 4.89 g) in dioxane (150 mL) and water (150 mL), Cs_2_CO_3_ (0.6 eq., 5.952 mmol, 1.939 g) was added. *trans-*2-Hexenal (1.2 eq., 11.904 mmol, 1.380 mL) in dioxane (20 mL) was further added dropwise over 10 h. The reaction mixture was stirred overnight at room temperature under argon atmosphere before adding the NaBH_4_ (2.5 eq., 25.80 mmol, 0.975 g) and 20 mL of water. After 2 h, the reaction mixture was acidified (pH 4) using a 2 M HCl solution. The solution was concentrated using a rotatory evaporator, and the solid was resolubilized in dichloromethane. The solution was filtrated and concentrated under reduce pressure to obtain a white powder (7.23 g), which was purified by flash chromatography (cyclohexane/ethyl acetate = 7/3, *v*/*v*) to obtain a slightly yellow crude mixture as a non-pure diastereoisomeric mixture (F) (2.43 g). The structure was confirmed by UPLC-ESI-MS (both diastereomers observed) and ^1^H NMR (300 MHz, CDCl_3_).

#### 3.3.4. Synthesis of *S*-3-(Hexan-1-ol)-*N*-(-l-γ-glutamyl)-l-cysteine Chloride (**G**)

In a non-pure diastereoisomeric solution of *S*-3-(hexan-1-ol)-*N*-(*N*-Boc-l-γ-glutamyl)-l-cysteine *tert*-butyl ester (**F**; 1 eq., 0.565 mmol, 318 mg) prepared in HCl-dioxane 4 M (2.6 mL), TIS (6 eq., 3.39 mmol, 0.631 mL) was added. The reaction mixture was stirred for 3 h at 0 °C, and then concentrated with a rotary evaporator to obtain a diastereoisomeric mixture *S*-3-(hexan-1-ol)-*N*-(-l-γ-glutamyl)-l-cysteine chloride (**G**). The structure was confirmed by UPLC-ESI-MS and ^1^H NMR (300 MHz, CDCl_3_).

### 3.4. Fermentation of Wort Spiked with γ-GluCys-3SHol

Wort was produced from pale malt (Pilsen, 6 RS) in a 60 L microbrewery Pilot Plant (Coenco, Oostkamp, Belgium). The 19.5 °Plato unhopped wort was obtained after 90 min of boiling and whirlpool clarification. In total, 250 mL of wort at 15 °P was pitched at 5 × 10^6^ living yeasts cells/mL and spiked with 15 mg/L of synthetic γ-GluCys-3SHol. Fermentation trials were conducted for 7 days at 24 °C and then 3 days at 4 °C under shaking at 100 rpm (Labwit ZWY-140 incubator shaker, Burwood East, Australia). For some experiments, fermentations were conducted at 12 °C or in a diluted wort of 6 °P.

### 3.5. Extraction of 3SHol from Fermented Spiked Media with Ag Cartridge

3SHol extraction from 150 mL fermented spiked media was performed according to Chenot et al. [14,29]. After NaCl saturation, 45 µL of 6SHol solution (6.7 mg/L prepared in EtOH) was added as an internal standard (IST, final concentration in beer = 2 µg/L). After shaking for 2 min, the remaining unsolubilized salt was removed. A liquid/liquid extraction of organic compounds with 50 mL of dichloromethane was performed, and the organic phase was recovered by centrifugation for 20 min at 4500 rpm and loaded on a Discovery^®^ Ag-Ion SPE Tube (Sigma-Aldrich: St. Louis, MO, USA). The cartridge was previously rinsed with 10 mL of dichloromethane and with 20 mL of acetonitrile. After reversing the cartridge, 10 mL of ultrapure water was passed over the cartridge before eluting the PFTs using 20 mL of washed cysteine solution (4 × 60 mL dichloromethane for washing 853 mg of cysteine in 80 mL of ultrapure water). A liquid/liquid extraction of the eluent was performed with bidistilled dichloromethane (5 mL for 5 min and 10 mL for 10 min). The organic phase was dried on anhydrous Na_2_SO_4_ and concentrated to 250 µL in a Danish–Kuderna distillation apparatus and to 70 µL on a Dufton column.

### 3.6. Gas Chromatography–Pulsed Flame Photometric Detection

One microliter (µL) of free thiol extract was analyzed with a Agilent 5973 N gas chromatograph from Agilent (Santa Clara, CA, USA) equipped with a splitless injector maintained at 250 °C. Compounds were analyzed with a wall-coated open tubular apolar CP-Sil5-CB capillary (50 m length, 0.32 mm i.d., and 1.2 µm film thickness). The carrier gas was He, and the pressure was set at 90 kPa. The oven temperature was programmed to increase from 36 to 85 °C at 20 °C per min, 145 °C at 1 °C per min, 220 °C at 3 °C per min, and finally held for 30 min at 220 °C. The column was connected to the OI Analytical PFPD detector (model 5380; combustor internal diameter: 2 mm) from Agilent (Santa Clara, CA, USA). The following parameters were selected for the PFPD detector: temperature, 220 °C; voltage, 590 V; gate width, 18 ms; gate delay, 6 ms; trigger level, 400 mV; and pulse frequency, 3.33 Hz. PFPD chromatograms were recorded throughout elution; MSD ChemStation F.01.03.2357 software (https://www.agilent.com/en/product/software-informatics/analytical-software-suite/chromatography-data-systems/openlab-chemstation, accessed on 10 February 2020) was used to process the resulting data. The following equation was used for 3SHA and 3SHol (X) quantitation (IST relative recovery factor set at 1):

$${µg\;L}^{-1}\;\mathrm{of}\;X={µg\;L}^{-1}\;\mathrm{of}\;\mathrm{IST}\;\times \frac{X\;\mathrm{area}}{\mathrm{IST}\;\mathrm{area}}\times \frac{\mathrm{IST}\;\mathrm{response}\;\mathrm{coefficient}}{X\;\mathrm{response}\;\mathrm{coefficient}}$$

### 3.7. Release Efficiency Determination

The release efficiency of 3SHol and 3SHA was calculated with the following equations:

$$3\mathrm{SHol}\;\mathrm{release}\;\mathrm{efficiency}\;\left(\%\right)=\frac{{µg\;L}^{-1}\;3\mathrm{SHol}}{{µg\;L}^{-1}\;\mathrm{added}\;\mathrm{bound}\;3\mathrm{SHol}}\times \frac{\mathrm{bound}\;3\mathrm{SHol}\;\mathrm{molar}\;\mathrm{weight}}{\mathrm{free}\;3\mathrm{SHol}\;\mathrm{molar}\;\mathrm{weight}}\times 100$$

$$3\mathrm{SHA}\;\mathrm{release}\;\mathrm{efficiency}\;\left(\%\right)=\frac{{µg\;L}^{-1}3\mathrm{SHA}}{{µg\;L}^{-1}\;\mathrm{added}\;\mathrm{bound}\;3\mathrm{SHol}}\times \frac{\mathrm{bound}\;3\mathrm{SHol}\;\mathrm{molar}\;\mathrm{weight}}{\mathrm{free}\;3\mathrm{SHA}\;\mathrm{molar}\;\mathrm{weight}}\times 100$$

The results are given as mean values of duplicates.

### 3.8. Statistical Analyses

All analytical measurements were carried out in duplicate. Multiple comparisons of the means were performed with Student–Newman–Keuls tests (JMP Program 17). Values sharing no common letter were significantly different (*p* < 0.05).

## 4. Conclusions

This study evaluated, through fermentation trials, the ability of eight *S. pastorianus* and three *S. cerevisiae* strains (including SafBrew^TM^ LA-01 var. *chevalieri*) to release the polyfunctional thiol from γ-GluCys-3SHol. Our findings emphasize the importance of both yeast strain and fermentation conditions (temperature and wort density). The lager yeasts BRAS-45 and SafLager^TM^ E-30 showed the highest release efficiencies (far above those previously measured for Cys-precursors). This suggests the existence, in these lager strains, of a β-lyase acting more efficiently on the dipeptidic precursor. BRAS-45 additionally showed significant esterification rates, enhancing its aromatic potential. In contrast, SafAle^TM^ K-97 had the lowest efficiency of release from the dipeptidic precursor, despite its unprecedented action on Cys-3SHol. Unexpectedly, although also active on the γ-GluCys-intermediate, SafBrew^TM^ LA-01 reached even higher release efficiency with G-3SHol. This suggests, for the first time, the possibility of direct release from the tripeptide (albeit less pronounced in a 6 °P wort). All these hypotheses should now be confirmed by monitoring each intermediate by UPLC-MS in the course of experimental fermentations. Deeper investigations are also needed to understand why reducing the fermentation temperature to 12 °C drastically decreased thiol release by BRAS-45, while the opposite was observed with SafLager^TM^ E-30.

## Data Availability

Data are contained within the article.

## Abbreviations

(PFTs) polyfunctional thiols; (*S.*) *Saccharomyces*; (γ-GluCys-) γ-Glutamylcysteine; (G-) glutathionylated; (Cys-) cysteinylated; (CysGly-) cysteinylglycine-; (°P) °Plato; (°C) °Celsius; (3SHol) 3-sulfanylhexanol; (3S4MPol) 3-sulfanyl-4-methylpentanol; (3SPol) 3-sulfanylpentanol; (γ-GT) γ-glutamyltranspeptidase; (CPY) carboxypeptidase; (GC) gas chromatography; (IST) internal standard; (PFPD) pulsed-flame photometric detection; (UPLC) ultra-performance liquid chromatography; (MS) mass spectroscopy; (ESI) electron spray ionization; (NMR) nuclear magnetic resonance.
